# Supplementary material for: Identification of photoperiod-induced specific miRNAs in the adrenal glands of Sunite sheep (Ovis aries)
Source: Front Vet Sci. 2022 Jul 22;9:888207. doi: 10.3389/fvets.2022.888207 (PMC9354845; doi:10.3389/fvets.2022.888207)
Supplement: Supplementary file 1 [file Data_Sheet_1.ZIP › Supplementary file/miRDeep2 file.zip!/miRDeep2 file/Know_Structure/SP42A3/oar-mir-3955.pdf]

miRBase precursor : oar-mir-3955
Total read count : 222
oar-miR-3955-5p read count : 190
oar-miR-3955-3p read count : 8
remaining reads : 24

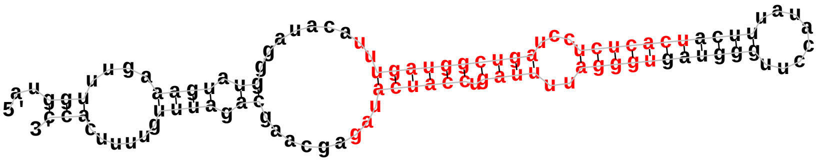

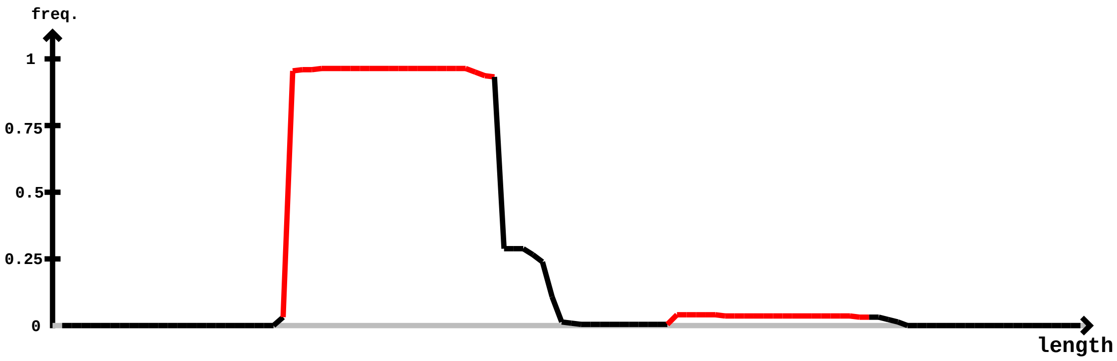


```
                                               oar-miR-3955-5p
                                                                                  oar-miR-3955-3p
5'- augguuugaaaguaugggauacauuugauggcugauccucucacuacuuuauaccuuggguagugggauuuuaguccaucauagagcaagcagauuuguuuucacc   -3'   exp
    ...........((((...))))...((((((((((...(((((((((((........)))))))))))..)))).)))))).(((((((......)))))))....    reads   mm   sample
    ......................auuugauggcugauccucucac..............................................................    1       0    seq
    ......................auuugauggcugauccucucacu.............................................................    3       0    seq
    ......................auuugauggcugauccucucacuacuu.........................................................    1       0    seq
    ......................auuugauggcugauccucucacuacuuu........................................................    2       0    seq
    .......................uuugauggcugauccucuc................................................................    3       0    seq
    .......................uuugauggcugauccucuca...............................................................    3       0    seq
    .......................uuugauggcugauccucucacu.............................................................    140     0    seq
    .......................uuugauggcugauccucucacuacu..........................................................    5       0    seq
    .......................uuugauggcugauccucucacuacuu.........................................................    5       0    seq
    .......................uuugauggcugauccucucacuacuuu........................................................    25      0    seq
    .......................uuugauggcugauccucucacuacuuua.......................................................    21      0    seq
    .......................uuugauggcugauccucucacuacuuuau......................................................    1       0    seq
    .......................uuugauggcugauccucucacuacuuuaua.....................................................    1       0    seq
    .......................uuugauggcugauccucucacuacuuuauaccuuggguaguggga......................................    1       0    seq
    ........................uugauggcugauccucucacuacuuu........................................................    1       0    seq
    ..........................gauggcugauccucucacuacuuu........................................................    1       0    seq
    ...............................................................ugggauuuuaguccaucau........................    1       0    seq
    ...............................................................ugggauuuuaguccaucauaga.....................    2       0    seq
    ...............................................................ugggauuuuaguccaucauagag....................    2       0    seq
    ...............................................................ugggauuuuaguccaucauagagc...................    3       0    seq
```
